# Enhancing Yields and Extending Production Cycles of Bacteriocin from the *Bacillus cereus* Group Through the Optimization of Culture Conditions and Removal of Proteolytic Digestion

**DOI:** 10.3390/microorganisms14010206

**Published:** 2026-01-16

**Authors:** Mengyu Gao, Jiajia Ding, Congyue Yang, Shu Liu, Huawei Zeng, Xin Zeng, Deyin Zhao, Bingyue Xin

**Affiliations:** Anhui Province Key Laboratory of Pollutant Sensitive Materials and Environmental Remediation, College of Life Sciences, Huaibei Normal University, Huaibei 235000, China

**Keywords:** *Bacillus cereus* group, bacteriocin, yield enhancement, production cycle extension, culture conditions, enzymatic hydrolysis

## Abstract

*Bacillus cereus* group strains are prolific producers of diverse bacteriocins with significant application potentials; however, their industrial utilization is often hampered by short production cycles and low yields. Using the leaderless bacteriocin toyoncin as a model, we systematically evaluated the impact of culture medium, temperature, and initial pH on its production. Our findings demonstrate that these factors critically affected yield, with no production under oligotrophic, acidic, or high-temperature conditions. Optimal production was achieved in MH medium (initial pH 8.5, 25 °C), significantly enhancing fermentation duration and yield compared to original conditions (LB medium, 30 °C, pH 7.0). Transcriptional analyses revealed that these improvements were attributable to extended transcription periods and increased transcript levels of the toyoncin gene cluster. Furthermore, we demonstrated that toyoncin disappearance in the supernatant is caused by transcriptional cessation and degradation by membrane-associated proteases. By combining optimized fermentation with protease inhibition, a high and stable toyoncin yield of 53.86 mg/L was achieved, representing a 3.07-fold increase over the initial yield (17.52 mg/L). This study establishes an integrated strategy to enhance bacteriocin production through simultaneous optimization of culture conditions and inhibition of enzymatic degradation, providing important insights for advancing bacteriocin development in the *Bacillus cereus* group.

## 1. Introduction

Bacteriocins are ribosomally synthesized antimicrobial peptides produced by bacteria [1]. They exhibit potent antimicrobial activity both in vivo and in vitro, along with advantages such as low or no toxicity, and ease of bioengineering [2]. These properties make bacteriocins highly promising for applications in diverse fields, including food preservation, medicine, and agriculture [3,4].

In recent years, more than twenty distinct types of bacteriocins have been identified in the *Bacillus cereus* group, including lantibiotics, circular bacteriocins, leaderless bacteriocins, sactipeptides, glycocins, thiazolylpeptides, and lasso peptides [5]. These bacteriocins exhibit diverse antimicrobial spectra and varying levels of antimicrobial activity. Some demonstrate broad-spectrum activity against multiple pathogenic bacteria—for instance, cerecidins show activity against a broad spectrum of Gram-positive bacteria, including vancomycin-resistant *Enterococcus faecalis* (VRE) and multidrug-resistant *Staphylococcus aureus* (MDRSA) [6]. Others are narrow-spectrum, targeting specific pathogens exclusively. Examples include thuricin CD, which is highly specific to *Clostridioides difficile* [7], and toyoncin, reported to selectively inhibit the foodborne pathogens *Listeria monocytogenes* and *B. cereus* [8]. These findings underscore the *Bacillus cereus* group bacteriocins’ broad potential for development and application across various fields.

However, we have noted that several reported bacteriocins from this group, such as bacicyclicin XIN-1, bacins, mycoidesin, and toyoncin, suffer from critical limitations—including short production cycles (typically confined to a narrow window of 4–8 h during the mid-logarithmic phase) and low yields (often reported below 20 mg/L in native producers) [5,8,9,10]. This transient and low-level production presents a major bottleneck: it not only complicates reliable extraction and scale-up but also severely limits the feasibility of obtaining sufficient material for downstream applications, such as detailed structure-activity relationship studies, formulation development, or preclinical trials. Consequently, this gap between discovery and practical deployment significantly hinders the translation of their promising in vitro activity into real-world solutions. Filling this gap by developing strategies to enhance yield and stabilize production is therefore of critical importance to unlock the full applied potential of these bacteriocins.

In this study, we focused on toyoncin—a representative leaderless bacteriocin from the *Bacillus cereus* group previously identified in our preliminary research, which exhibits the typical short production cycle (approximately 5 h) and low yield (~17 mg/L under standard LB conditions) [8]. We hypothesized that the low and transient production of toyoncin arises from a dual limitation: (1) suboptimal transcriptional activation of its biosynthetic gene cluster under initial fermentation conditions; (2) rapid post-synthetic degradation by host proteases. To address this, we proposed an integrated strategy that combines systematic fermentation optimization to maximize biosynthesis with targeted inhibition of degradation pathways. We further aimed to elucidate the underlying molecular mechanisms linking environmental parameters to gene expression and product stability. We initiated this study by systematically evaluating the effects of culture media, initial pH, and temperature on bacteriocin production, thereby establishing conditions unfavorable for bacteriocin production and the optimal fermentation condition. Through transcriptional analysis of the biosynthetic gene cluster, we elucidated the molecular basis for enhanced production yield and prolonged synthesis duration under optimized conditions. Furthermore, we identified the critical role of membrane-associated proteases in bacteriocin degradation. By synergistically combining optimized fermentation parameters with protease inhibition, we ultimately achieved a further improvement in the production cycle and yield of toyoncin. We expect that this combined approach will not only significantly extend the production window and increase the final titer of toyoncin but also establish a generalizable methodological framework for overcoming similar production bottlenecks in other bioactive bacterial metabolites.

## 2. Materials and Methods

### 2.1. Evaluation of Toyoncin Production Under Diverse Culture Conditions

Toyoncin production was assessed under varying liquid culture media, initial pH values, and fermentation temperatures. Ten distinct media were evaluated: Luria–Bertani (LB) broth, Linden Grain (LG) broth, Brain Heart Infusion (BHI) broth, Tryptic Soy Broth (TSB), Mueller–Hinton (MH) broth, Nutrient Broth (NB), Reasoner’s 2A (R2A) broth, and 1:10 dilutions of LB, MH, and TSB. Among these, LB, LG, BHI, TSB, MH, and NB were designated as nutrient-rich media, whereas R2A, 1/10 LB, 1/10 MH, and 1/10 TSB were used as nutrient-poor media. The initial pH of each medium was adjusted to 5.5, 7.0, and 8.5 using 1 M HCl or NaOH. These pH values were selected to cover acidic, neutral, and alkaline conditions commonly encountered in bacterial fermentation and to investigate the pH tolerance of the toyoncin biosynthetic system. A single colony of *Bacillus toyonensis* XIN-YC13 was inoculated into LB broth (initial pH 7.0) and incubated at 30 °C for 12 h to prepare the seed culture. The cells were then harvested by centrifugation, washed, and resuspended in sterile physiological saline. This bacterial suspension was used to inoculate the test media at 2% (*v*/*v*). The inoculated media were incubated at temperatures ranging from 20 to 40 °C (20, 25, 30, 35, and 40 °C). This temperature range spans from sub-optimal to supra-optimal growth temperatures for *B. cereus* group strains, allowing us to assess the thermosensitivity of toyoncin production [11,12,13,14]. The cultivation lasted 36 h, with samples collected at 2 h intervals to monitor bacterial growth and toyoncin production. Bacterial growth was monitored by measuring the optical density at 600 nm (OD_600_). The antimicrobial activity of the cell-free supernatant, indicative of toyoncin production, was determined against *B. cereus* ATCC 14579 using an agar diffusion assay [8]. Briefly, the indicator strain was mixed into molten soft agar at a final concentration of approximately 5 × 10^5^ CFU/mL and poured into plates. After solidification, wells were punched and filled with the supernatant. The plates were first refrigerated at 4 °C for 2 h to allow pre-diffusion, then incubated at 30 °C for 16 h, after which the diameters of the inhibition zones were measured.

### 2.2. Analysis of toyA Gene Transcription Under Two Culture Conditions

*Bacillus toyonensis* XIN-YC13 was cultivated for 36 h under two distinct conditions: (1) LB medium, initial pH 7.0, 30 °C; and (2) MH medium, initial pH 8.5, 25 °C. Bacterial samples (1 mL) were collected at hourly intervals, and the cells were harvested by centrifugation at 12,000× *g* for 5 min. Total RNA was extracted from the cell pellets using a Spin Column Bacteria Total RNA Purification Kit (Sangon Biotech, Shanghai, China), according to the manufacturer’s instructions. Subsequent cDNA synthesis was performed using the MightyScript Plus First Strand cDNA Synthesis Master Mix (Sangon Biotech, China). Quantitative real-time PCR (qPCR) was carried out on an ABI Prism 7500 instrument (Applied Biosystems, Foster City, CA, USA) using RealStar Universal SYBR qPCR Mix (GenStar, Beijing, China). The transcriptional profile of the structural gene *toyA* was analyzed with the specific primers ToyAF (5′-ACGGTACAAAAGCATACAATGTT-3′) and ToyAR (5′-TCGATTAATCCAGCAGCTTTT-3′). The 16S rRNA gene, amplified with primers 16SF (5′-GAAGGCGACTTTCTGGTCTG-3′) and 16SR (5′-CCTTTGAGTTTCAGCCTTGC-3′), served as the internal reference gene. The relative changes in *toyA* mRNA expression levels were calculated using the comparative 2^−ΔΔCt^ method.

### 2.3. Verification of the Proteolytic Degradation of Toyoncin by Membrane-Associated Proteases of Bacillus toyonensis XIN-YC13

*Bacillus toyonensis* XIN-YC13 was inoculated into two sets of nine flasks each, containing either LB medium (pH 7.0) or MH medium (pH 8.5). Cultures in LB medium were incubated at 30 °C, while those in MH medium were incubated at 25 °C, both with shaking. For each medium, the experimental procedure was as follows: At predetermined time points (9 h for LB cultures and 26 h for MH cultures), a protease inhibitor cocktail (P1026, Beyotime Biotechnology, Haimen, China; composition: 23 mM AEBSF, 2 mM Bestatin, 0.3 mM E64, and 2 mM Pepstatin A in DMSO) was added to three of the flasks, which were then incubated further until 36 h. Simultaneously, cells from three additional flasks were harvested by centrifugation (12,000× *g*, 10 min, 4 °C). The resulting cell-free supernatants were transferred to three new flasks, which continued to be incubated with shaking until 36 h. The remaining three flasks served as untreated controls. Throughout the 0 to 36 h cultivation period, samples were collected from all nine flasks in each set at 1 h intervals. All samples were centrifuged to remove bacterial cells. The antimicrobial activity of the resulting supernatants against the indicator strain *B. cereus* ATCC 14579 was determined using a well diffusion assay, as previously described.

### 2.4. Quantification of Toyoncin Production

The yield of toyoncin was determined as follows: The crude extract (CE) of toyoncin was prepared as described by Wang et al. [8]. Briefly, cultures of strain XIN-YC13 under different conditions were centrifuged (12,000× *g*, 10 min) to collect the supernatants. One liter of the supernatant was adsorbed using 200 g of 7HP resin, followed by washing with 500 mL of water and 300 mL of 30% ethanol. Elution was performed using 80% ethanol (pH 2.0). The eluate was then concentrated using a rotary evaporator to obtain 10 mL of the crude toyoncin extract. The toyoncin content in the crude extract was determined by high-performance liquid chromatography (HPLC) using the external standard method. The preparation of pure toyoncin was carried out as described by Wang et al. [8]. The pure toyoncin was dissolved in ddH_2_O to prepare standard solutions with concentrations of 10 mg/L, 100 mg/L, 1000 mg/L, and 10,000 mg/L. A standard curve was plotted with the concentration of the standard solution (C) as the abscissa and the corresponding average peak area (A) as the ordinate, yielding the regression equation: A = *k*C + b. The concentration of the target substance in the test sample solution was calculated by substituting the peak area of the test sample solution into the regression equation of the standard curve. The content of toyoncin in the crude extract obtained through final calculation was then converted to determine the toyoncin content in 1 L of fermentation supernatant. Representative HPLC chromatograms are provided in Supplementary Figure S9.

## 3. Results

### 3.1. Evaluation of Bacteriocin Toyoncin Production Across Varying Liquid Media, Initial pH, and Fermentation Temperatures

**Figure 1:**
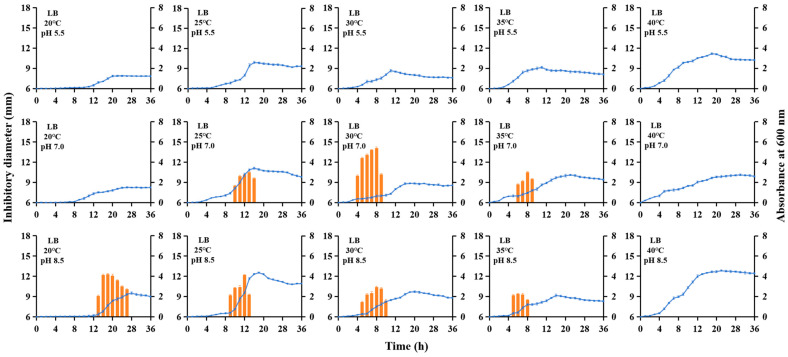
Kinetics of toyoncin produced by *Bacillus toyonensis* XIN-YC13 in LB medium under varying initial pH and fermentation temperatures. The optical density of the YC13 culture was measured at 600 nm (●). The antimicrobial activity of the supernatant of *B. toyonensis* XIN-YC13 against *B. cereus* ATCC 14579 under different culture conditions was presented as the diameter of the inhibition zone (orange bars).

**Figure 2:**
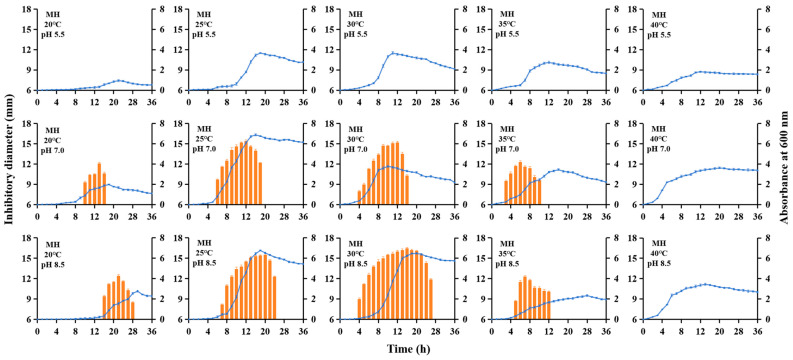
Kinetics of toyoncin produced by *Bacillus toyonensis* XIN-YC13 in MH medium under varying initial pH and fermentation temperatures. The optical density of the XIN-YC13 culture was measured at 600 nm (●). The antimicrobial activity of the supernatant of *B. toyonensis* XIN-YC13 against *B. cereus* ATCC 14579 under different culture conditions was presented as the diameter of the inhibition zone (orange bars).

The XIN-YC13 strain was tested for the production of leaderless bacteriocin toyoncin under ten liquid media, three initial pH levels, and five fermentation temperatures. The results showed that in four oligotrophic media (R2A, 1/10 LB, 1/10 TSB, and 1/10 MH), no toyoncin was produced regardless of temperature or initial pH (Supplementary Figures S1–S4). In contrast, the other six nutrient-rich media supported toyoncin production under certain combinations of temperature and initial pH (Figure 1 and Figure 2 and Supplementary Figures S5–S8). Additionally, no bacteriocin was produced under all high-temperature cultivation conditions (40 °C) or at an initial pH of 5.5. Compared to the original cultivation conditions (LB medium, 30 °C, initial pH 7.0), which only supported a 6 h production cycle with a moderate yield (a maximum inhibition zone diameter of 14.2 mm at 8 h), the MH medium at 25 °C and an initial pH of 8.5 exhibited a substantially extended production cycle (4–26 h, lasting 23 h) and a significantly higher yield (reaching a maximum inhibition zone diameter of 16.6 mm at 16 h) (Figure 1 and Figure 2).

### 3.2. Transcription Duration and Transcription Level of the toyA Gene Under Different Culture Conditions

**Figure 3:**
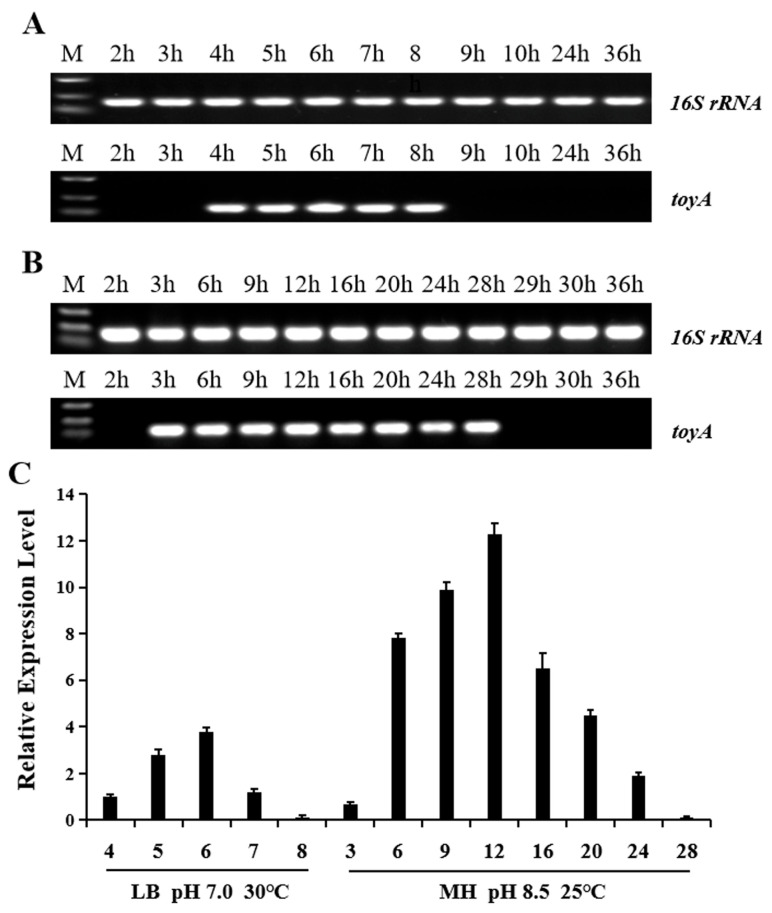
Comparative analysis of transcription duration and levels at different time points for the *toyA* gene in the toyoncin gene cluster under two culture conditions. (**A**) Transcription duration analysis of *toyA* gene under LB medium at 30 °C with initial pH 7.0 (original fermentation condition). (**B**) Transcription duration analysis of *toyA* gene under MH medium at 25 °C with initial pH 8.5 (high-producing condition). (**C**) Comparative analysis of *toyA* gene transcription levels under two culture conditions. The transcription level of gene *toyA* at 4 h in LB medium at 30 °C with initial pH 7.0 was set as the 1-fold reference against which levels under other fermentation conditions were compared.

Transcriptional profiles of the key structural gene *toyA* in the toyoncin biosynthetic gene cluster were comparatively analyzed under two distinct culture conditions: LB medium at 30 °C with initial pH 7.0 (original fermentation conditions), and MH medium at 25 °C with initial pH 8.5 (high-producing condition). As depicted in Figure 3A, when cultured in LB medium (30 °C, initial pH 7.0), transcription of *toyA* was observed from 4 h to 8 h, with a total transcription duration of 5 h (Figure 3B). In contrast, under the condition of MH medium (25 °C, initial pH 8.5), *toyA* transcription was detected from 3 h to 28 h, resulting in a transcription duration of 26 h.

Furthermore, we compared the transcriptional level of the *toyA* gene at different time points under these two culture conditions. As shown in Figure 3C, the transcriptional level of *toyA* under both culture conditions exhibited an initial increase followed by a decrease. In the MH medium (25 °C, initial pH 8.5), the transcript level peaked at 12 h, whereas in the LB medium (30 °C, initial pH 7.0), it peaked at 6 h. The peak level under the former condition was 3.25-fold higher than that under the latter.

### 3.3. The Membrane-Associated Protease Degrades the Bacteriocin in the Fermentation Supernatant

**Figure 4:**
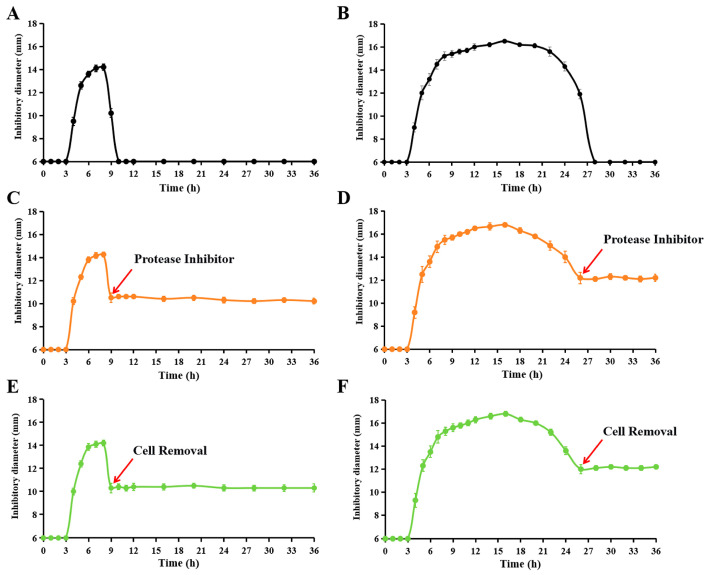
The proteolytic activity responsible for toyoncin degradation is membrane-associated and not secreted into the culture supernatant. (**A**,**B**) Production of toyoncin by strain XIN-YC13 under two culture conditions: (**A**) LB medium, initial pH 7.0, 30 °C; (**B**) MH medium, initial pH 8.5, 25 °C. (**C**,**D**) Verification of toyoncin degradation in XIN-YC13 cultures following the addition of a protease inhibitor cocktail (P1026, Beyotime Biotechnology) at 9 h ((**C**), LB medium) and 26 h ((**D**), MH medium). (**E**,**F**) Assessment of toyoncin degradation in cell-free fermentation supernatant of strain XIN-YC13 under (**E**) LB and (**F**) MH culture conditions. Red arrows in all panels indicate the time of protease inhibitor addition or cell removal.

Under all bacteriocin-producing conditions, the yield in the fermentation supernatant gradually decreases after reaching a plateau and eventually diminishes entirely (Figure 1 and Figure 2 and Supplementary Figures S5–S8). We hypothesized that this decrease resulted from proteolytic degradation by either membrane-associated or secreted proteases. To test this hypothesis, a protease inhibitor cocktail was added to XIN-YC13 cultures when degradation was evident. As shown in Figure 4A, when strain XIN-YC13 was cultured in LB medium at an initial pH of 7.0 and 30 °C, the antimicrobial activity of its supernatant peaked at 6 h of fermentation, decreased by 8 h, and was completely lost by 10 h. However, when protease inhibitors were added to the culture at 9 h, the antimicrobial activity of the fermentation supernatant remained stable until the end of the assay (36 h). A similar trend was observed under MH medium conditions with an initial pH of 8.5 and 25 °C: the antimicrobial activity of the supernatant of strain XIN-YC13 peaked at 16 h of fermentation, began to decline at 18 h, and was completely absent by 28 h. In contrast, when protease inhibitors were added at 26 h, the antimicrobial activity of the fermentation supernatant remained unchanged until the end of the assay (36 h). These results indicate that proteases in the fermentation broth mediate the degradation of the bacteriocin toyoncin.

To further distinguish whether bacteriocin degradation was caused by proteases in the supernatant or membrane-associated proteases, we removed cells from the culture and monitored the antimicrobial activity of the resulting cell-free supernatant. As shown in Figure 4C,D, the antimicrobial activities of the supernatants from the two culture conditions both remained stable after cell removal. This demonstrates that the proteolytic activity for toyoncin is membrane-associated rather than secreted into the culture supernatant.

### 3.4. Determination of Yield of Toyoncin Under Optimal Fermentation Conditions with Protease Inhibitor Supplementation

**Figure 5:**
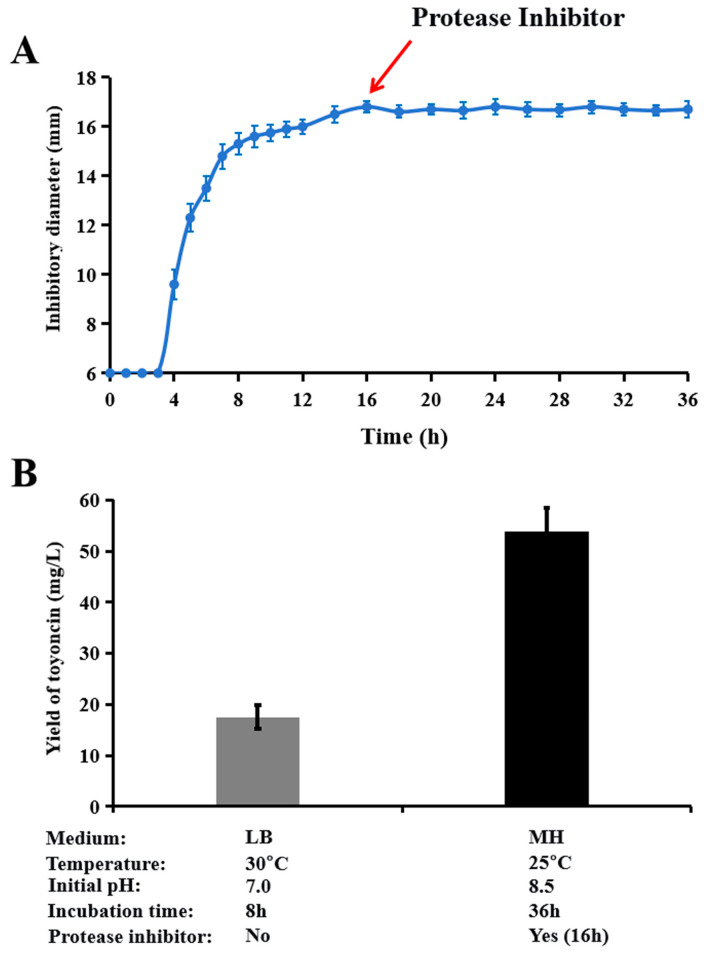
Determination of yield of toyoncin under optimal fermentation conditions with protease inhibitor supplementation. (**A**) Determination of the production period of toyoncin when a protease inhibitor was added to the culture of XIN-YC13 strain. A protease inhibitor cocktail (P1026, Beyotime Biotechnology) was added to the bacterial culture at 16 h of incubation. (**B**) Determination of toyoncin yield under two fermentation conditions. Data are presented as mean ± standard deviation (n = 3).

Based on the determined optimal fermentation conditions for bacteriocin toyoncin (MH medium at 25 °C with an initial pH of 8.5) and the suppression of toyoncin degradation by proteases in the culture (achieved by adding a protease inhibitor at 16 h of fermentation, the time point of peak toyoncin production), we characterized the production period and final yield of toyoncin. As shown in Figure 5A, after the addition of the protease inhibitor at 16 h, the antimicrobial activity of the fermentation supernatant remained stable until the end of the assay (36 h). Furthermore, a 3.07-fold increase in toyoncin yield was achieved, reaching 53.86 ± 4.56 mg/L at 36 h (mean ± SD, n = 3), compared to 17.52 ± 2.32 mg/L under the initial conditions (LB medium, 30 °C, pH 7.0, 8 h) (Figure 5B and Supplementary Figure S9).

## 4. Discussion

In this study, we investigated the production cycle and yield of toyoncin under different culture conditions and found that no toyoncin was detected under any of the oligotrophic conditions tested (Supplementary Figures S1–S4). We hypothesize that this phenomenon is primarily governed by the tight metabolic regulation of bacteriocin synthesis in response to nutrient availability. Bacteriocin production is not only an energy-intensive process, imposing a significant metabolic burden on the producer [15], but it is also a typical “luxury trait” whose expression is strategically regulated. Under nutrient-limited (oligotrophic) conditions, bacterial cells prioritize resource allocation towards essential growth and maintenance functions over the synthesis of non-essential secondary metabolites [16]. This resource reallocation likely leads to the repression of the toyoncin biosynthesis gene cluster, as supporting such a costly process would be disadvantageous for survival and competition in a low-nutrient environment [17]. In addition, toyoncin was not synthesized under acidic (pH 5.5) or high-temperature (40 °C) conditions (Figure 1 and Figure 2 and Supplementary Figures S5–S8). *B. toyonensis* exhibits optimal growth under neutral to slightly alkaline pH conditions, and when cultured at pH 5.5, the bacterium is subjected to acidic stress [11,12,13,14,18]. Under such stress conditions, the cells likely activate a global “stress response program,” diverting cellular resources and transcriptional machinery to pathways essential for sustaining life (e.g., pH homeostasis, glycolysis, tricarboxylic acid cycle, and protein repair), while simultaneously downregulating dispensable pathways, including secondary metabolite synthesis [19]. Therefore, we speculate that the functional proteins involved in toyoncin synthesis may also become inactivated or their expression suppressed under these stressful conditions. Furthermore, since 40 °C is not a heat stress for *B. toyonensis* but rather the upper limit of its optimal growth temperature, we hypothesize that the toyoncin gene cluster is tightly regulated and remains unexpressed at this temperature, possibly due to temperature-sensitive regulatory mechanisms that have yet to be elucidated. We did not test temperatures above 40 °C, such as 45 °C, as these could cause severe heat shock and significantly suppress overall metabolism, thereby making bacteriocin production impossible.

In previous studies, it has been observed that several bacteriocins from the *B. cereus* group are not produced by their native strains and were instead identified and characterized through in vitro biosynthesis strategy. For example, since *cerM* (essential for modification) was not expressed under LB conditions, preventing cerecidin detection, the authors achieved production by co-expressing *cerA* and *cerM* in *E. coli* [6]. This approach generated precerecidins, which were then proteolytically processed to yield mature cerecidins. Our study provides a potential explanation for this phenomenon: researchers may not have identified the suitable culture conditions—such as medium composition, temperature, and pH—required for bacteriocin production. Thus, this study offers a critical implication for future research: in culture-based studies aimed at identifying novel bacteriocins from *B. cereus* group strains and optimizing bacteriocin yields, it is advisable to test a variety of nutrient-rich media, as well as different temperatures and pH values. Such an approach could maximize the potential to induce and enhance the expression of bacteriocin biosynthetic gene clusters, thereby improving the efficiency of discovering and characterizing novel bacteriocins and effectively increasing their production. While more granular media optimization (e.g., specific carbon/nitrogen sources) and AI-based approaches are valuable future directions, our broad screening of commercially available complex media provides a crucial and practical first step to identify promising base conditions.

Among the various fermentation conditions tested, MH medium at 25 °C with an initial pH of 8.5 was identified as the optimal condition for toyoncin production. This optimized condition resulted in a significant increase in bacteriocin yield compared to the initial condition (LB medium at 30 °C with initial pH 7.0). The deliberate comparison of these two compositionally distinct, yet commonly used complex media (LB vs. MH) was instrumental, revealing not only a quantitative difference in yield but, more importantly, a fundamental qualitative difference in the production physiology. Specifically, the production duration was extended from 6 h to 28 h. Further investigation revealed that this enhancement is directly linked to both the prolonged transcription duration and the increased transcript levels of the bacteriocin synthetase gene cluster. This finding is pivotal because it shifts the paradigm for optimizing bacteriocin production from a purely empirical nutrient-tuning approach to a mechanism-guided strategy. Our results confirm a crucial direction for future research: to increase yields, strategies should focus on extending the transcription duration and boosting the transcript levels of the target gene cluster. We consider that systematic optimization of specific carbon and nitrogen sources remains a vital future step for maximizing titer, our work provides the essential mechanistic target for such optimization. Future media engineering efforts should rationally select and balance nutrients that specifically support this prolonged, high-level transcriptional state in *B. toyonensis*. In this context, the MH medium serves as a promising baseline formulation for developing such a rationally designed, high-performance production medium.

Beyond the culture condition optimization employed in this study, we will also focus on the influence of transcriptional regulators within the bacteriocin gene cluster in further research. Numerous studies have reported that various types of regulatory regulators, such as global transcriptional regulators located outside bacteriocin synthesis gene clusters, internal two-component regulatory systems, and positive or negative regulators within the clusters, control bacteriocin production [20,21,22,23,24,25,26,27,28,29]. Within the toyoncin gene cluster, three putative regulatory proteins have been identified [8]; however, their specific roles—whether they act as negative or positive regulators—remain unclear. In future work, we aim to experimentally determine the regulatory functions of these proteins on bacteriocin production. Subsequently, we plan to leverage this knowledge to enhance yields, potentially through knockout of negative regulators or overexpression of positive regulators. Additionally, we intend to explore other strategies to boost toyoncin production, such as the addition of specific inducers, microbial co-cultivation, and the synthetic biology approach of reconstructing the biosynthetic pathway. These methods have been widely reported to activate the expression and enhance the production of various antimicrobial compounds [30,31,32,33,34,35,36]. We will also attempt to apply these approaches to improve the production of bacteriocins from the *B. cereus* group.

Several reported bacteriocins from the *B. cereus* group exhibit a transient presence in the culture supernatant, disappearing shortly after secretion [5,8,9,10]. The underlying mechanism for this disappearance, however, had remained elusive. This study specifically investigated this phenomenon and revealed, for the first time, that membrane-associated proteases are responsible for bacteriocin degradation, representing the primary cause of its clearance from the supernatant. Notably, both the optimization of fermentation conditions and the addition of protease inhibitors significantly extended the production duration and substantially increased the yield of toyoncin (Figure 4B). Bioinformatic analysis identified ten genes encoding putative membrane-associated proteases in the strain XIN-YC13 genome (Table S1). Currently, it remains unclear which specific protease(s) mediate the degradation. Future work will employ gene knockout strategies to systematically evaluate the contribution of each protease. The primary significance of this effort lies in its potential to prevent the degradation of synthesized bacteriocin, thereby facilitating its stable accumulation in the supernatant and prolonging the production window. It should be noted that the cocktail of protease inhibitors used here is unsuitable for large-scale industrial production due to cost constraints. Therefore, the identification and subsequent knockout of the key gene(s) encoding the responsible protease(s) in the native strain, to permanently eliminate bacteriocin degradation during fermentation, constitutes a major objective of our future research. This genetic engineering approach would offer a more scalable and cost-effective solution for industrial production than chemical inhibition.

## 5. Conclusions

Bacteriocins from the *Bacillus cereus* group hold significant potential, but their development is often hampered by short production cycles and low yields. This study focused on toyoncin, a leaderless bacteriocin, and systematically optimized its production. We identified culture conditions that either completely inhibit or significantly enhance toyoncin yield. The optimized fermentation protocol not only increased the yield but also extended the production window. Mechanistic investigations revealed that this improvement was linked to a prolonged transcription duration and a substantial increase in the transcript levels of the toyoncin synthetic gene cluster. Furthermore, we identified that membrane-associated proteases, rather than secreted ones, are responsible for toyoncin degradation. By integrating optimized fermentation with protease inhibition, a high and stable yield of 53.86 mg/L was achieved. To our knowledge, this is the first in-depth study on the production characteristics of a bacteriocin from the *B. cereus* group. The strategies and insights presented here provide a crucial foundation for enhancing the production and discovery of novel bacteriocins. Specifically, the use of a defined medium (MH), the precise timing of protease inhibition, and the mechanistic understanding of degradation collectively outline a scalable and industrially relevant production framework. The achieved yield under controlled conditions demonstrates strong potential for pilot-scale fermentation. Future efforts should focus on translating these laboratory protocols into bioreactor operations, conducting techno-economic analyses, and implementing the proposed genetic strategies (e.g., regulator modulation and protease gene knockout) to further improve efficiency and reduce costs, thereby fully realizing the commercial potential of toyoncin and related bacteriocins.

## Data Availability

The original contributions presented in this study are included in the article/Supplementary Material. Further inquiries can be directed to the corresponding author.

## Supplementary Materials

The following supporting information can be downloaded at: https://www.mdpi.com/article/10.3390/microorganisms14010206/s1, Table S1: Putative membrane-associated proteases encoded in the genome of strain XIN-YC13. Figure S1: Kinetics of toyoncin produced by *Bacillus toyonensis* XIN-YC13 in R2A medium under varying initial pH and fermentation temperatures. The optical density of the YC13 culture was measured at 600 nm (●). The antimicrobial activity of the supernatant of *B. toyonensis* YC13 against *B. cereus* ATCC14579 under different culture conditions was presented as the diameter of the inhibition zone. No toyoncin was produced regardless of temperature or initial pH with R2A medium. Figure S2: Kinetics of toyoncin produced by *Bacillus toyonensis* XIN-YC13 in 1/10 LB medium under varying initial pH and fermentation temperatures. The optical density of the YC13 culture was measured at 600 nm (●). The antimicrobial activity of the supernatant of *B. toyonensis* YC13 against *B. cereus* ATCC14579 under different culture conditions was presented as the diameter of the inhibition zone. No toyoncin was produced regardless of temperature or initial pH with 1/10 LB medium. Figure S3: Kinetics of toyoncin produced by *Bacillus toyonensis* XIN-YC13 in 1/10 TSB medium under varying initial pH and fermentation temperatures. The optical density of the YC13 culture was measured at 600 nm (●). The antimicrobial activity of the supernatant of *B. toyonensis* YC13 against *B. cereus* ATCC14579 under different culture conditions was presented as the diameter of the inhibition zone. No toyoncin was produced regardless of temperature or initial pH with 1/10 TSB medium. Figure S4: Kinetics of toyoncin produced by *Bacillus toyonensis* XIN-YC13 in 1/10 MH medium under varying initial pH and fermentation temperatures. The optical density of the YC13 culture was measured at 600 nm (●). The antimicrobial activity of the supernatant of *B. toyonensis* YC13 against *B. cereus* ATCC14579 under different culture conditions was presented as the diameter of the inhibition zone. No toyoncin was produced regardless of temperature or initial pH with 1/10 MH medium. Figure S5: Kinetics of toyoncin produced by *Bacillus toyonensis* XIN-YC13 in BHI medium under varying initial pH and fermentation temperatures. The optical density of the XIN-YC13 culture was measured at 600 nm (●). The antimicrobial activity of the supernatant of *B. toyonensis* XIN-YC13 against *B. cereus* ATCC14579 under different culture conditions was presented as the diameter of the inhibition zone (orange bars). Figure S6: Kinetics of toyoncin produced by *Bacillus toyonensis* XIN-YC13 in LG medium under varying initial pH and fermentation temperatures. The optical density of the XIN-YC13 culture was measured at 600 nm (●). The antimicrobial activity of the supernatant of *B. toyonensis* XIN-YC13 against *B. cereus* ATCC14579 under different culture conditions was presented as the diameter of the inhibition zone (orange bars). Figure S7: Kinetics of toyoncin produced by *Bacillus toyonensis* XIN-YC13 in NB medium under varying initial pH and fermentation temperatures. The optical density of the XIN-YC13 culture was measured at 600 nm (●). The antimicrobial activity of the supernatant of *B. toyonensis* XIN-YC13 against *B. cereus* ATCC14579 under different culture conditions was presented as the diameter of the inhibition zone (orange bars). Figure S8: Kinetics of toyoncin produced by *Bacillus toyonensis* XIN-YC13 in TSB medium under varying initial pH and fermentation temperatures. The optical density of the XIN-YC13 culture was measured at 600 nm (●). The antimicrobial activity of the supernatant of *B. toyonensis* XIN-YC13 against *B. cereus* ATCC14579 under different culture conditions was presented as the diameter of the inhibition zone (orange bars). Figure S9: Determination of toyoncin yield under different fermentation conditions. (A) HPLC analysis of toyoncin standards at varying concentrations (10.0, 100.0, 1000.0, and 10,000.0 mg/L). Each sample was analyzed with a constant injection volume of 50 μL. (B) HPLC profile of crude toyoncin extract obtained from fermentation in LB medium at 30 °C and initial pH 7.0, harvested at 8 h. (C) HPLC profile of crude toyoncin extract from fermentation in MH medium at 25 °C and initial pH 8.5, harvested at 36 h. To suppress protease-mediated degradation, a protease inhibitor was added at 16 h, corresponding to the peak production time of toyoncin. Crude extracts in (B) and (C) were each prepared from 1 L of fermentation broth by adsorption onto macroporous resin, followed by elution and concentration to a final volume of 10 mL. The injection volume for HPLC analysis was 50 μL. In all chromatograms, the peak corresponding to toyoncin is marked with a red arrow. All experiments were performed in triplicate, and representative HPLC chromatograms are shown here.

## Abbreviations

The following abbreviations are used in this manuscript:

LB	Luria–Bertani
LG	Linden Grain
BHI	Brain Heart Infusion
TSB	Tryptic Soy Broth
MH	Mueller–Hinton
NB	Nutrient Broth
R2A	Reasoner’s 2A
HPLC	high-performance liquid chromatography
CE	crude extract
