# Supplementary material for: Enhancing Yields and Extending Production Cycles of Bacteriocin from the Bacillus cereus Group Through the Optimization of Culture Conditions and Removal of Proteolytic Digestion
Source: Microorganisms. 2026 Jan 16;14(1):206. doi: 10.3390/microorganisms14010206 (PMC12844061; doi:10.3390/microorganisms14010206)
Supplement: Supplementary file 1 [file microorganisms-14-00206-s001.zip › microorganisms-4020941-supplementary.pdf]

Table S1 Putative membrane-associated proteases encoded in the genome of strain XIN-YC13.

GenBank ID	Number of amino acids	Proposed fuction
NSL65436.1	613	S8 family serine peptidase
NSL66697.1	549	M4 family metallopeptidase
NSL68189.1	549	M4 family metallopeptidase
NSL66875.1	890	M4 family metallopeptidase
NSL67111.1	397	S8 family serine peptidase
NSL67424.1	740	M60 family metallopeptidase
NSL68111.1	546	S8 family serine peptidase
NSL68170.1	764	M60 family metallopeptidase
NSL68817.1	269	C39 family peptidase
NSL69631.1	391	S1C family serine protease

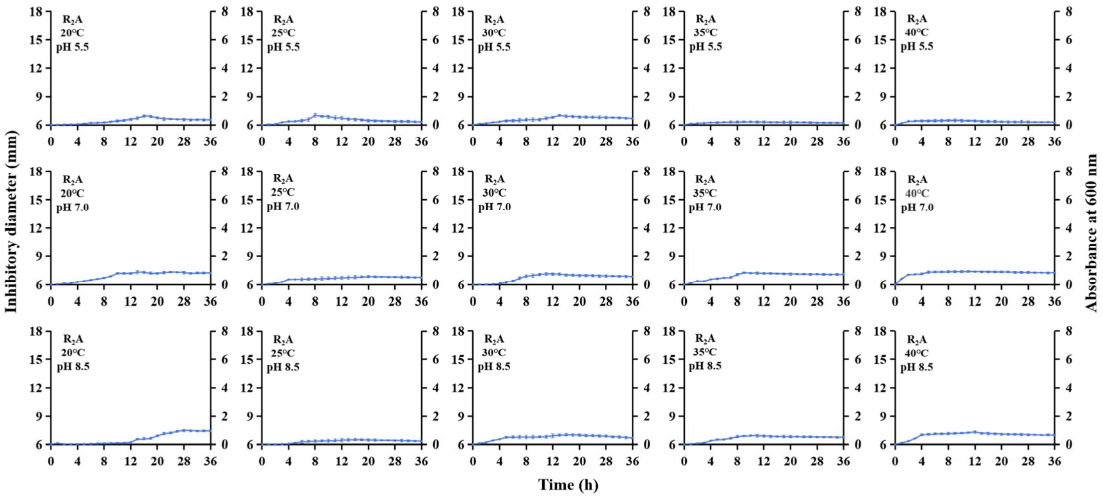

Figure S1 Kinetics of toyoncin produced by *Bacillus toyonensis* XIN-YC13 in R2A medium under varying initial pH and fermentation temperatures. The optical density of the YC13 culture was measured at 600 nm (●). The antimicrobial activity of the supernatant of *B. toyonensis* YC13 against *B. cereus* ATCC14579 under different culture conditions was presented as the diameter of the inhibition zone. No toyoncin was produced regardless of temperature or initial pH with R2A medium.

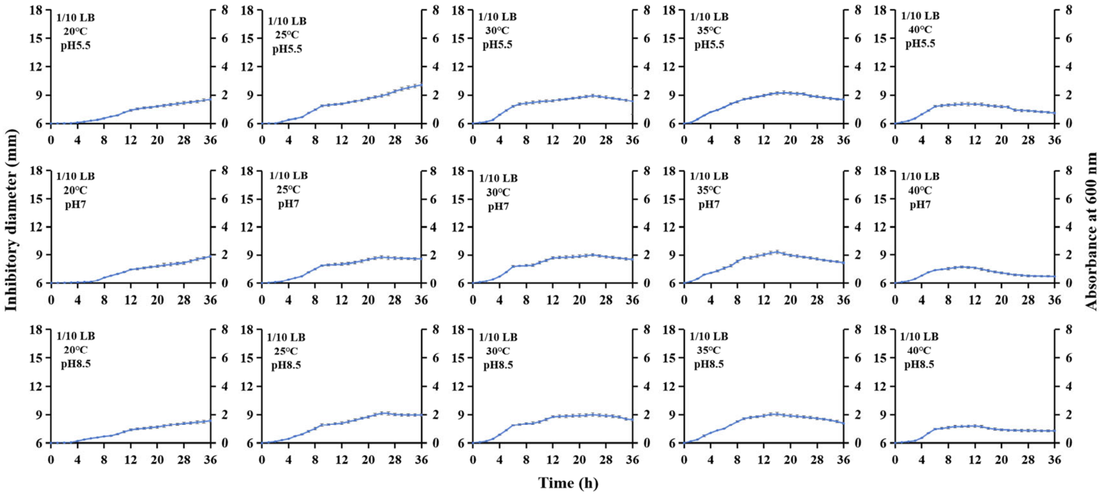

Figure S2 Kinetics of toyoncin produced by *Bacillus toyonensis* XIN-YC13 in 1/10 LB medium under varying initial pH and fermentation temperatures. The optical density of the YC13 culture was measured at 600 nm (●). The antimicrobial activity of the supernatant of *B. toyonensis* YC13 against *B. cereus* ATCC14579 under different culture conditions was presented as the diameter of the inhibition zone. No toyoncin was produced regardless of temperature or initial pH with 1/10 LB medium.

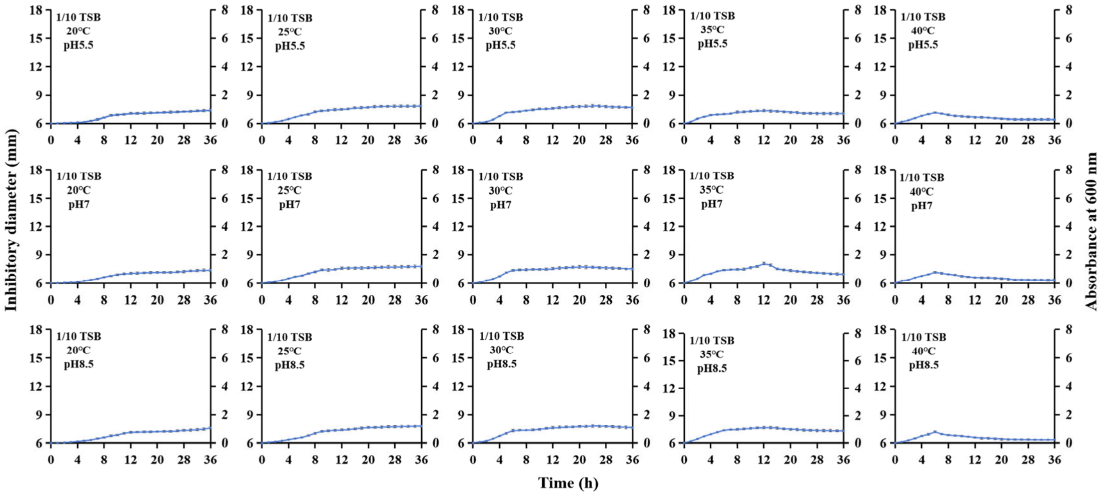

Figure S3 Kinetics of toyoncin produced by *Bacillus toyonensis* XIN-YC13 in 1/10 TSB medium under varying initial pH and fermentation temperatures. The optical density of the YC13 culture was measured at 600 nm (●). The antimicrobial activity of the supernatant of *B. toyonensis* YC13 against *B. cereus* ATCC14579 under different culture conditions was presented as the diameter of the inhibition zone. No toyoncin was produced regardless of temperature or initial pH with 1/10 TSB medium.

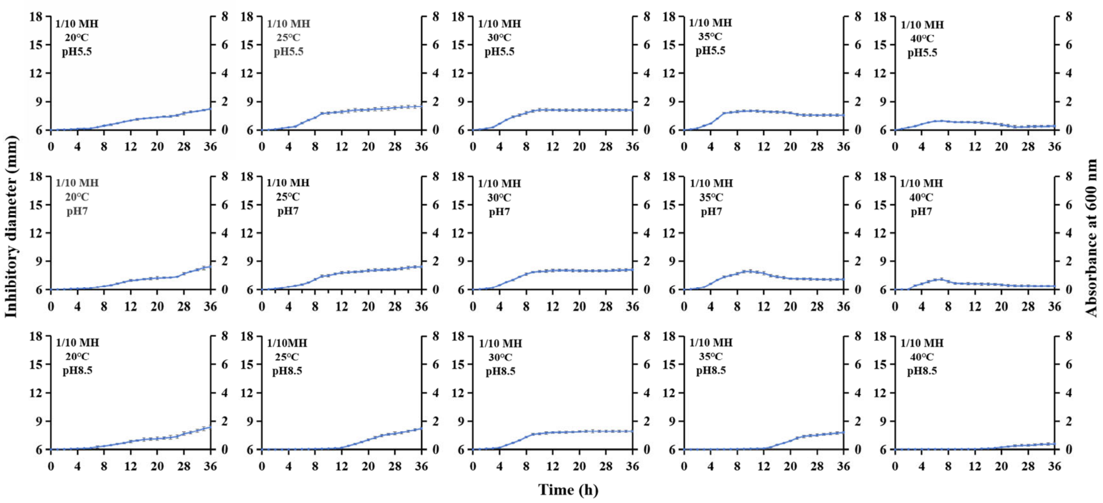

Figure S4 Kinetics of toyoncin produced by *Bacillus toyonensis* XIN-YC13 in 1/10 MH medium under varying initial pH and fermentation temperatures. The optical density of the YC13 culture was measured at 600 nm (●). The antimicrobial activity of the supernatant of *B. toyonensis* YC13 against *B. cereus* ATCC14579 under different culture conditions was presented as the diameter of the inhibition zone. No toyoncin was produced regardless of temperature or initial pH with 1/10 MH medium.

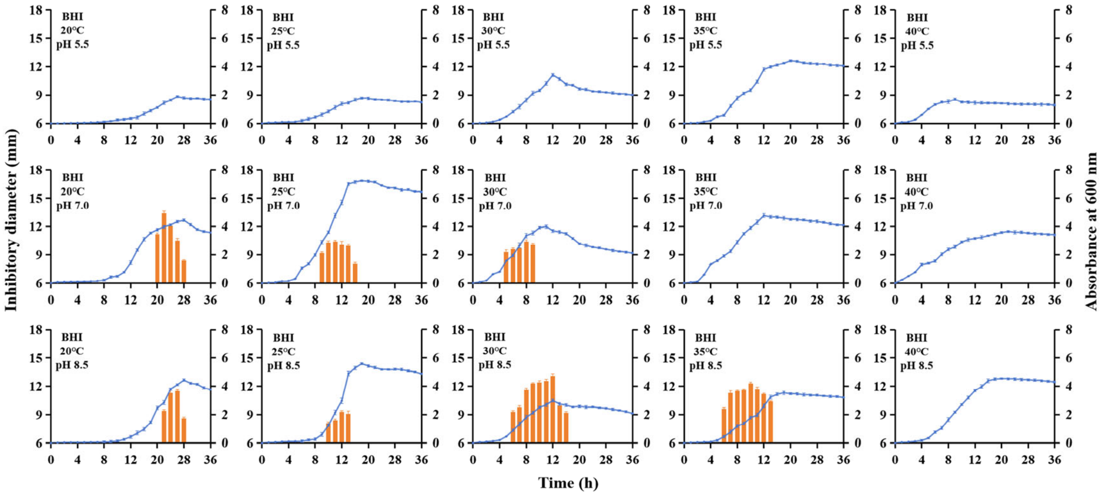

Figure S5 Kinetics of toyoncin produced by *Bacillus toyonensis* XIN-YC13 in BHI medium under varying initial pH and fermentation temperatures. The optical density of the XIN-YC13 culture was measured at 600 nm (●). The antimicrobial activity of the supernatant of *B. toyonensis* XIN-YC13 against *B. cereus* ATCC14579 under different culture conditions was presented as the diameter of the inhibition zone (orange bars).

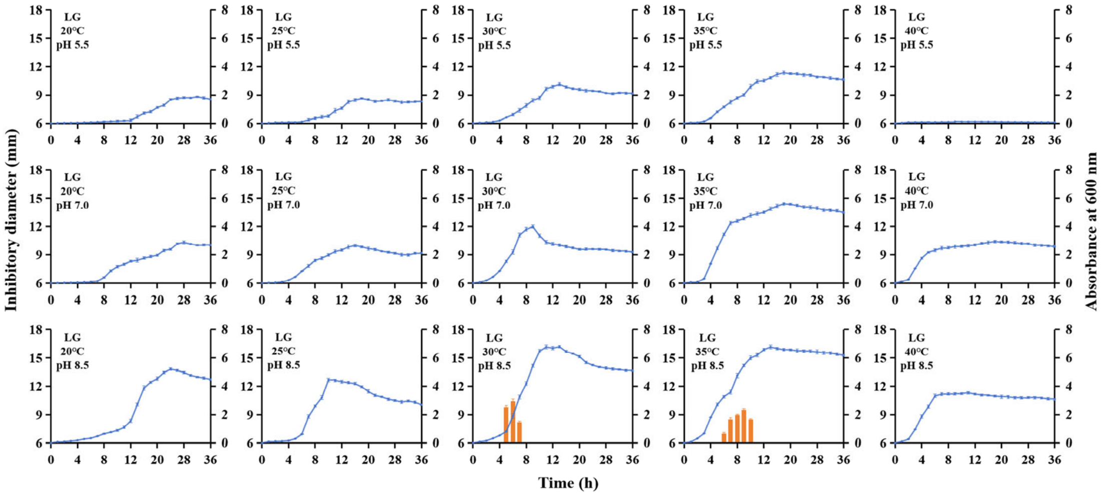

Figure S6 Kinetics of toyoncin produced by *Bacillus toyonensis* XIN-YC13 in LG medium under varying initial pH and fermentation temperatures. The optical density of the XIN-YC13 culture was measured at 600 nm (●). The antimicrobial activity of the supernatant of *B. toyonensis* XIN-YC13 against *B. cereus* ATCC14579 under different culture conditions was presented as the diameter of the inhibition zone (orange bars).

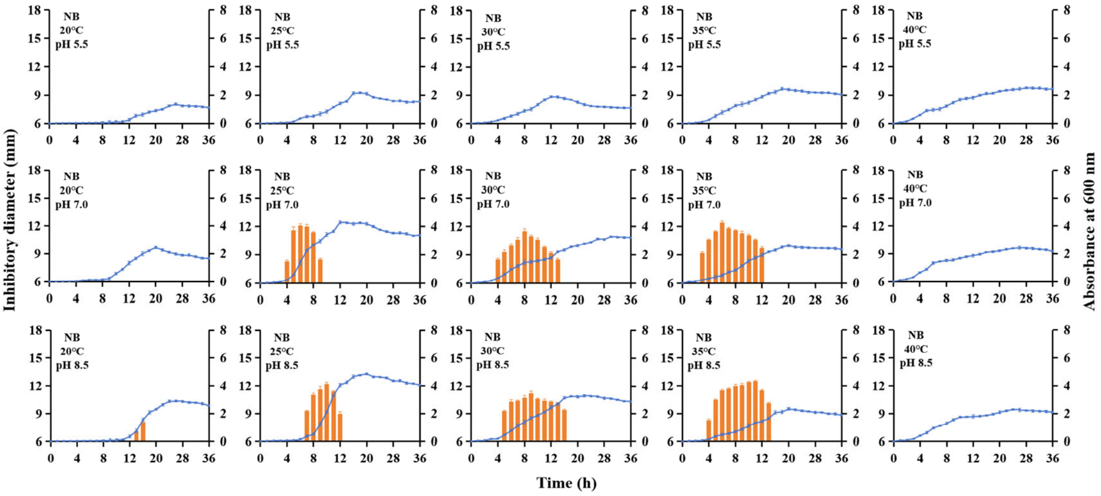

Figure S7 Kinetics of toyoncin produced by *Bacillus toyonensis* XIN-YC13 in NB medium under varying initial pH and fermentation temperatures. The optical density of the XIN-YC13 culture was measured at 600 nm (●). The antimicrobial activity of the supernatant of *B. toyonensis* XIN-YC13 against *B. cereus* ATCC14579 under different culture conditions was presented as the diameter of the inhibition zone (orange bars).

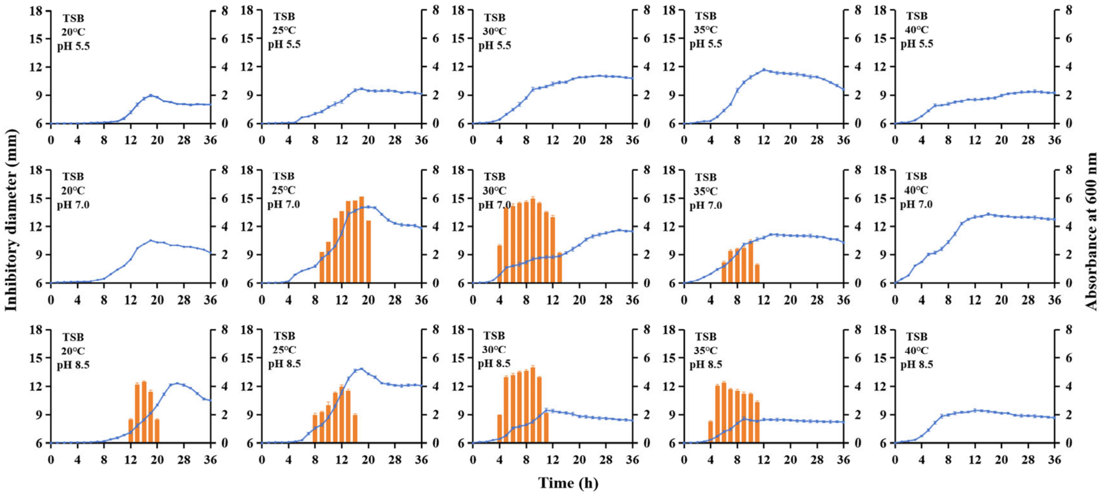

Figure S8 Kinetics of toyoncin produced by *Bacillus toyonensis* XIN-YC13 in TSB medium under varying initial pH and fermentation temperatures. The optical density of the XIN-YC13 culture was measured at 600 nm (●). The antimicrobial activity of the supernatant of *B. toyonensis* XIN-YC13 against *B. cereus* ATCC14579 under different culture conditions was presented as the diameter of the inhibition zone (orange bars).

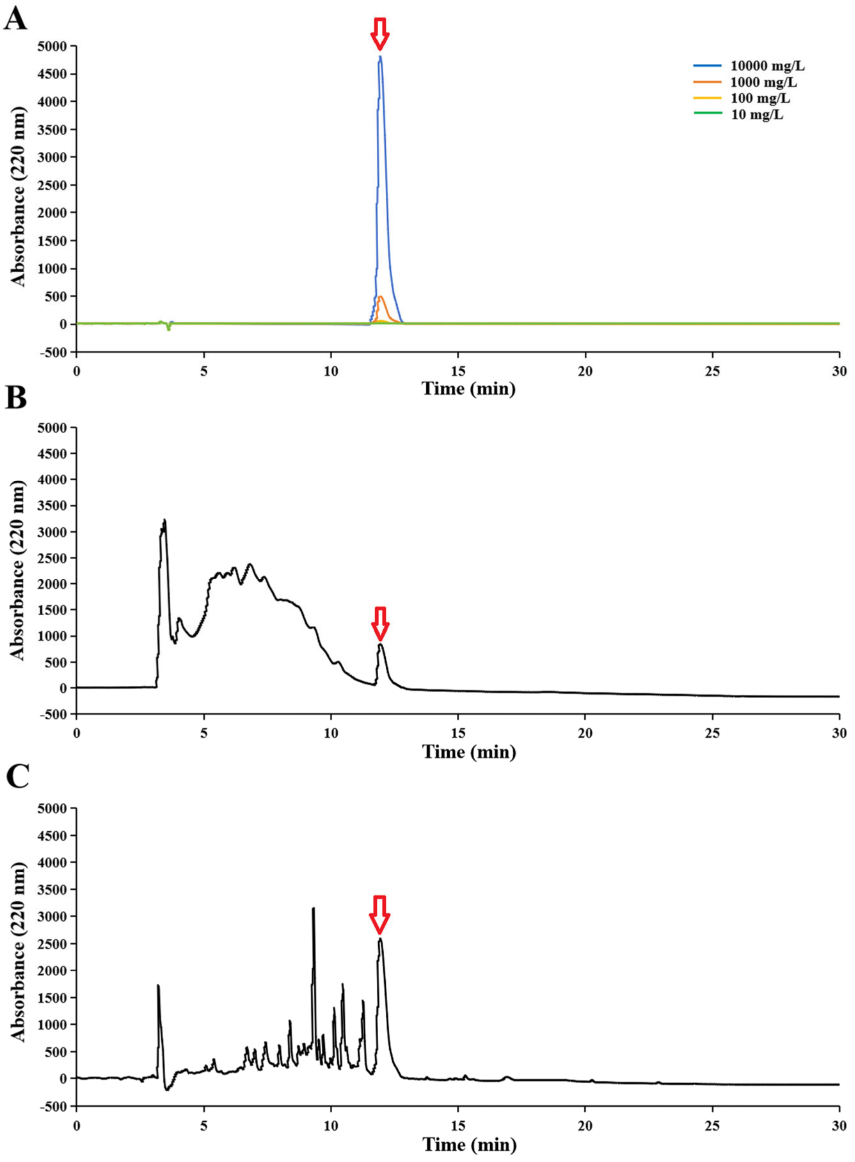

Figure S9 Determination of toyoncin yield under different fermentation conditions. (A) HPLC analysis of toyoncin standards at varying concentrations (10.0, 100.0, 1,000.0, and 10,000.0 mg/L). Each sample was analyzed with a constant injection volume of 50 μL. (B) HPLC profile of crude toyoncin extract obtained from fermentation in LB medium at 30°C and initial pH 7.0, harvested at 8 h. (C) HPLC profile of crude toyoncin extract from fermentation in MH medium at 25°C and initial pH 8.5, harvested at 36 h. To suppress protease-mediated degradation, a protease inhibitor was added at 16 h, corresponding to the peak production time of toyoncin. Crude extracts in (B) and (C) were each prepared from 1 L of fermentation broth by adsorption onto macroporous resin, followed by elution and concentration to a final volume of 10 mL. The injection volume for HPLC analysis was 50 μL. In all chromatograms, the peak corresponding to toyoncin is marked with a red arrow. All experiments were performed in triplicate, and representative HPLC chromatograms are shown here.
